# Impaired Coronary and Renal Vascular Function in Spontaneously Type 2 Diabetic Leptin-Deficient Mice

**DOI:** 10.1371/journal.pone.0130648

**Published:** 2015-06-22

**Authors:** Helena U. Westergren, Julia Grönros, Suvi E. Heinonen, Tasso Miliotis, Karin Jennbacken, Alan Sabirsh, Anette Ericsson, Ann-Cathrine Jönsson-Rylander, Sara Svedlund, Li-Ming Gan

**Affiliations:** 1 Department of Molecular and Clinical Medicine, Institute of Medicine, Sahlgrenska Academy at the University of Gothenburg, Gothenburg, Sweden; 2 CVMD iMED, AstraZeneca R&D Mölndal, Mölndal, Sweden; 3 Department of Clinical Physiology, Sahlgrenska University Hospital, Gothenburg, Sweden; University of Edinburgh, UNITED KINGDOM

## Abstract

**Background:**

Type 2 diabetes is associated with macro- and microvascular complications in man. Microvascular dysfunction affects both cardiac and renal function and is now recognized as a main driver of cardiovascular mortality and morbidity. However, progression of microvascular dysfunction in experimental models is often obscured by macrovascular pathology and consequently demanding to study. The obese type 2 diabetic leptin-deficient (ob/ob) mouse lacks macrovascular complications, i.e. occlusive atherosclerotic disease, and may therefore be a potential model for microvascular dysfunction. The present study aimed to test the hypothesis that these mice with an insulin resistant phenotype might display microvascular dysfunction in both coronary and renal vascular beds.

**Methods and Results:**

In this study we used non-invasive Doppler ultrasound imaging to characterize microvascular dysfunction during the progression of diabetes in ob/ob mice. Impaired coronary flow velocity reserve was observed in the ob/ob mice at 16 and 21 weeks of age compared to lean controls. In addition, renal resistivity index as well as pulsatility index was higher in the ob/ob mice at 21 weeks compared to lean controls. Moreover, plasma L-arginine was lower in ob/ob mice, while asymmetric dimethylarginine was unaltered. Furthermore, a decrease in renal vascular density was observed in the ob/ob mice.

**Conclusion:**

In parallel to previously described metabolic disturbances, the leptin-deficient ob/ob mice also display cardiac and renal microvascular dysfunction. This model may therefore be suitable for translational, mechanistic and interventional studies to improve the understanding of microvascular complications in type 2 diabetes.

## Introduction

Type 2 diabetes is recognized as a disease of chronic hyperglycemia that has reached epidemic scale, known to have the risk of affecting multiple organs including the kidneys, retina and heart, as well as peripheral extremities [1]. It is well established that type 2 diabetes patients have an increased incidence of cardiovascular (CV) complications and CV mortality. Furthermore, diabetes is also the number one cause of chronic kidney disease (CKD) and this condition increases the risk for CV disease even further [2,3]. In addition, type 2 diabetes patients suffer from impaired microvascular function [4] which now is recognised as an important mechanism underlying unfavourable CV outcome [5]. Microvascular function, as measured by coronary flow reserve (CFR), is impaired in type 2 diabetic patients with angiographically normal coronary arteries [6,7]. Furthermore the parallel progression of cardiac and renal vascular dysfunction in type 2 diabetes is a growing area of interest and importance. Clinically, coronary dysfunction is related to early renal dysfunction in terms of microalbuminuria in type 2 diabetic patients without obstructive coronary artery disease [4] as well as to decreased renal function at an early stage [8]. These clinical observations indeed further support microvascular impairment to be a common mechanism underlying both cardiac and renal vascular complications in type 2 diabetes.

From a translational perspective, there is a great need for animal models of diabetes-related microvascular complications for mechanistic as well as preclinical proof of principle studies. Vascular endothelial-dependent relaxation, coronary function and renal microvascular perfusion have been shown to be impaired in several models of type 2 diabetes [9–15]. Although the obese insulin resistant leptin deficient (ob/ob) mouse model is a well characterized non-insulin dependent type 2 diabetes model [16] the impact of diabetes progression on in vivo coronary and renal vascular function is not well understood. To study *in vivo* cardiac and renal vascular function, non-invasive techniques are crucial for longitudinal follow-up. The measurement of microvascular dysfunction by coronary flow velocity reserve (CFVR) in rodents can be assessed non-invasively by transthoracic color Doppler echocardiography in non-atherosclerotic models [17], and we have developed a protocol for mice [18]. Pulsatility index (PI) and resistive index (RI), derived from renal Doppler flow velocity profiles [19], can be used as tools for diagnosing renal artery stenosis, as well as markers for renal vascular function in e.g diabetic nephropathy in humans [20]. Furthermore, high renal PI and RI seem to be an independent predictor of a decline in estimated glomerular filtration rate in heart failure patients [21] as well as of CV survival [22]. In addition, RI has been measured using Doppler ultrasound in a mouse model of cardiorenal syndrome [23]. Thus, in this study, we aimed to use established Doppler ultrasound techniques to document parallel changes in cardiac and renal vascular function.

The ob/ob mouse strain is an obesity and insulin resistant model lacking atherosclerosis formation [24] and might therefore be useful for studies of microvascular dysfunction. The impact of pre-diabetes and early diabetes on cardiac and renal microvascular dysfunction is a growing area of interest. From that aspect, this model might appear more useful in the purpose of studying pathology related to early diabetic changes, since diabetes progression is not as severe as in other models, e.g. db/db mice. The current study therefore aimed to test the hypothesis that parallel early cardiac and renal microvascular dysfunction is prevalent in this non-atherosclerotic insulin resistant model, and that this is caused by microvascular structural and functional changes related to the vascular nitric oxide pathway.

## Material and Methods

The supporting ARRIVE Guidelines checklist is available as supporting information, see S1 ARRIVE Checklist.

### Animal Model

The study was performed in obese, insulin resistant and progressively type 2 diabetic homozygous male C57Bl/6J-*lep* ^*ob*^ mice (ob/ob, n = 12) and age matched lean litter mates (+/?) were used as healthy controls (lean, n = 12), (Jackson Laboratory, USA). Animals had free access to water and standard rodent chow diet (R3, Lantmännen, Stockholm, Sweden) in temperature-controlled facilities with a 12-h light and 12-h dark cycle, at 21–22°C. Mice were housed in Marcolon Polycarbonate cages either 4 animals/cage (ob/ob) or one animal/cage (lean) and acclimatized for 2 weeks before entering the study. All cages were environmentally enriched with sawdust, nest pads, gnaw sticks and egg cartoon. Mice were anesthetized in the imaging laboratory by inhalation of isoflurane gas (2.5%, Abbott Scandinavia, Solna, Sweden) due to its beneficial properties of quick onset and short half-life and thereby minimized anesthesia time. In our study we examined two groups (lean and ob/ob mice) which were not randomized due to the difference in phenotype. Furthermore, due to the obvious phenotype, blinding was not possible when performing the imaging. However, every other lean and ob/ob mouse was examined by ultrasound to minimize time shift. Blood samples were collected from the tail veins following each ultrasound scanning and mice put back in there home cages. Mice were sacrificed at 21 weeks of age during anesthesia (5% isoflurane) following blood withdrawal from the left ventricle. Furthermore, heart and kidneys were collected and weighed. A 4 mm thick slice of heart tissue at the level of mitral valve and both whole kidneys were collected and fixed in formalin for subsequent analysis of smaller vessels. All flow velocities (described below) were determined from signals that were stable for at least three consecutive heart beats and representative of the average of two cardiac cycles. Measurements off-line were done blinded to the examiner. Three mice were pre-terminated due to either tail infection (one ob/ob and one lean) or poor health condition (one ob/ob).

### Ethics Statement

The study was approved by the Regional Ethical Committee for Laboratory Animal Experiments of the University of Gothenburg in Sweden. All procedures conformed to the guidelines from Directive 2010/63/EU of the European Parliament on the protection of animals used for scientific purposes. All ultrasound examinations and surgery was performed under anesthesia, and all efforts were made to minimize suffering.

### Cardiac ultrasound and coronary flow velocity reserve (CFVR) protocol

**Fig 1:**
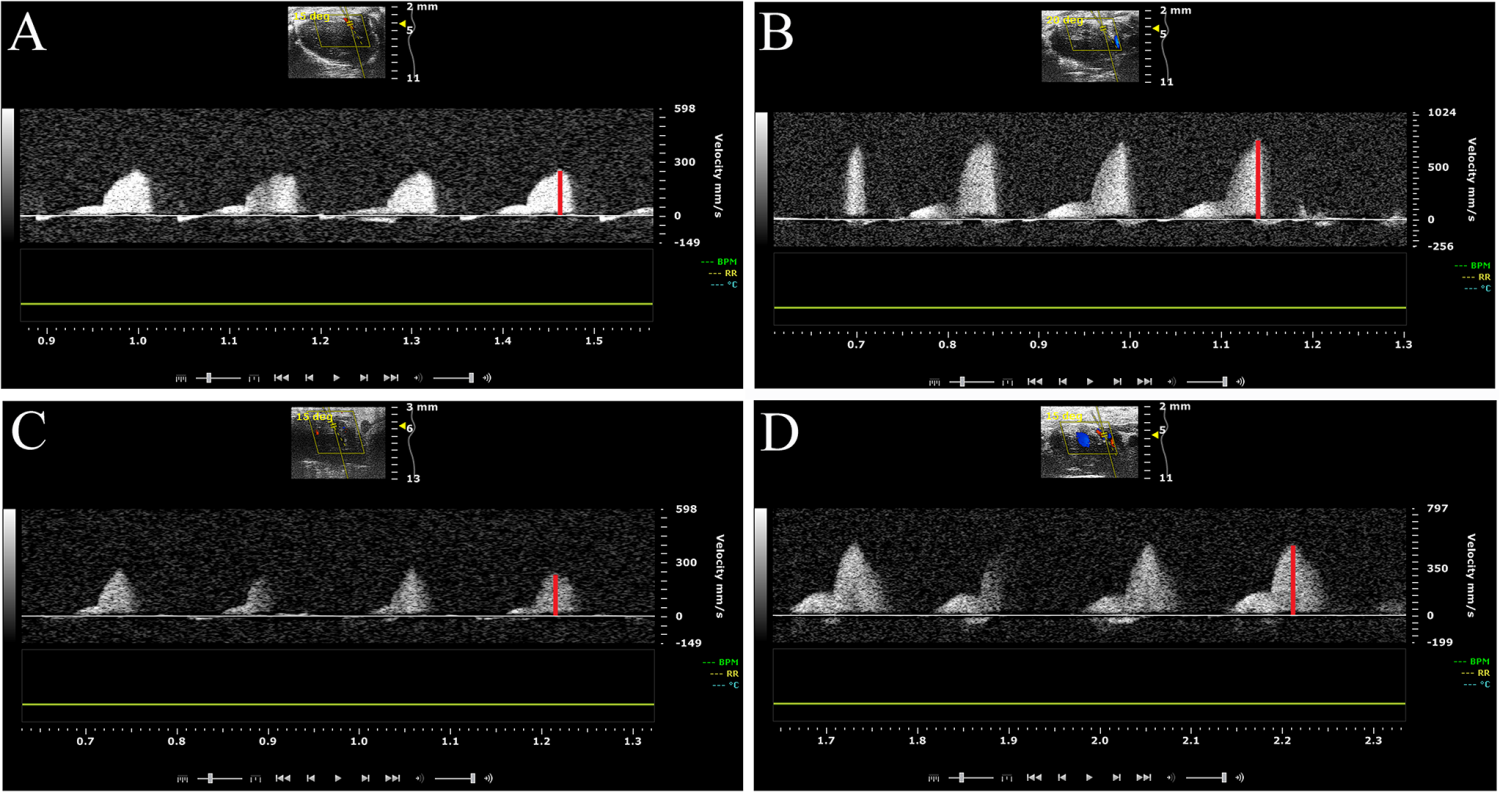
Representative color Doppler image for measurement of coronary flow Velocities. Typical recordings of resting and hyperaemic flow velocity measurement in the left coronary artery were performed with color Doppler ultrasound in lean and leptin-deficient (ob/ob) mice at 10, 16 and 21weeks of age. Hypereamic flow velocity was induced by intravenous infusion of adenosine (140 μg/kg/min). Coronary flow velocity reserve was calculated as the ratio of peak diastolic flow velocities (red line) before (resting) and during (hyperaemic) adenosine infusion. A: Resting coronay flow velocity in lean mice. B: Hyperaemic coronary flow velocity in lean mice. C: Resting coronary flow velocity in ob/ob mice. D: Hyperaemic coronary flow velocity in ob/ob mice.

Transthoracic echocardiography was performed at 10, 16 and 21 weeks of age in all mice using a high-frequency ultrasound imaging system (Vevo 2100 VisualSonics, Inc, Toronto, Ontario, Canada) with a 40-MHz central frequency transducer. The protocol has been described previously [18]. Briefly, mice were initially anesthetized with 2.5% isoflurane and kept on low levels (1.0–1.5%) during ultrasound examination. The total time of anesthesia and protocol performance was approximately 25 minutes/mouse and projections were captured in the same order in all mice. Isoflurane is known to have vasodilating effect at levels of 2.5% and the low levels of 1.0–1.5% have been shown hemodynamically neutral and thus used during all ultrasound scanning [25]. Isoflurane is widely used for ultrasound examination due to its rapid onset and short half-life, which enables good control of anesthesia time and depth. These advantages are of particular importance in longitudinal studies to minimize stress levels in the mice. Moreover, our current CFVR protocol was validated during isoflurane anesthesia which has also been shown to give rise to stable heart rate [18,26]. Mice were kept on a ventilated and heated bench. The chest was shaved using an electrical razor and hair removal cream. A catheter (0.4 x 10mm, Becton Dickinson Infusion Therapy, Helsingborg, Sweden) was inserted into the tail vein for intravenous infusion of 0.25 mg/ml adenosine (140 μg/kg/min) (ITEM Development AB, Stocksund, Sweden) administration, using an infusion pump. Mean volume infused at 21 weeks of age were 17.4 μl/min and 28.3 μμl/min, for lean and ob/ob mice respectively. This dose of adenosine has been validated previously to induce hyperaemia without influencing systemic hemodynamics [18]. Resting and hyperaemic flow velocity in the left coronary artery (LCA) was measured in a modified long-axis view, recorded with a pulsed-wave Doppler in proximal LCA (Fig 1). Measurements of left ventricle dimensions were performed in standard B-mode images in short axis views at the papillary level.

Fractional shortening (FS) was calculated as ((LVEDD—LVESD) / LVEDD) * 100

where LVEDD is the left ventricle end-diastolic diameter and LVESD the left ventricle end-systolic diameter.

Left ventricle mass (LVM) was calculated as (LVEDD + AW + PW)^3^ –(LVEDD)^3^

where AW and PW are the anterior and posterior wall thicknesses in the left ventricle, respectively.

Heart rate was calculated as average from three consecutive cardiac cycles in Doppler mode.

CFVR was calculated as the ratio of peak diastolic flow velocities before and during adenosine infusion: CFVR = Hyperemic coronary flow velocity / Basal coronary flow velocity. We have previously shown that the coefficient of variation for intra- and interobserver regarding peak CFVR are 5.6% and 7.7%, respectively [27].

### Renal ultrasound protocol

**Fig 2:**
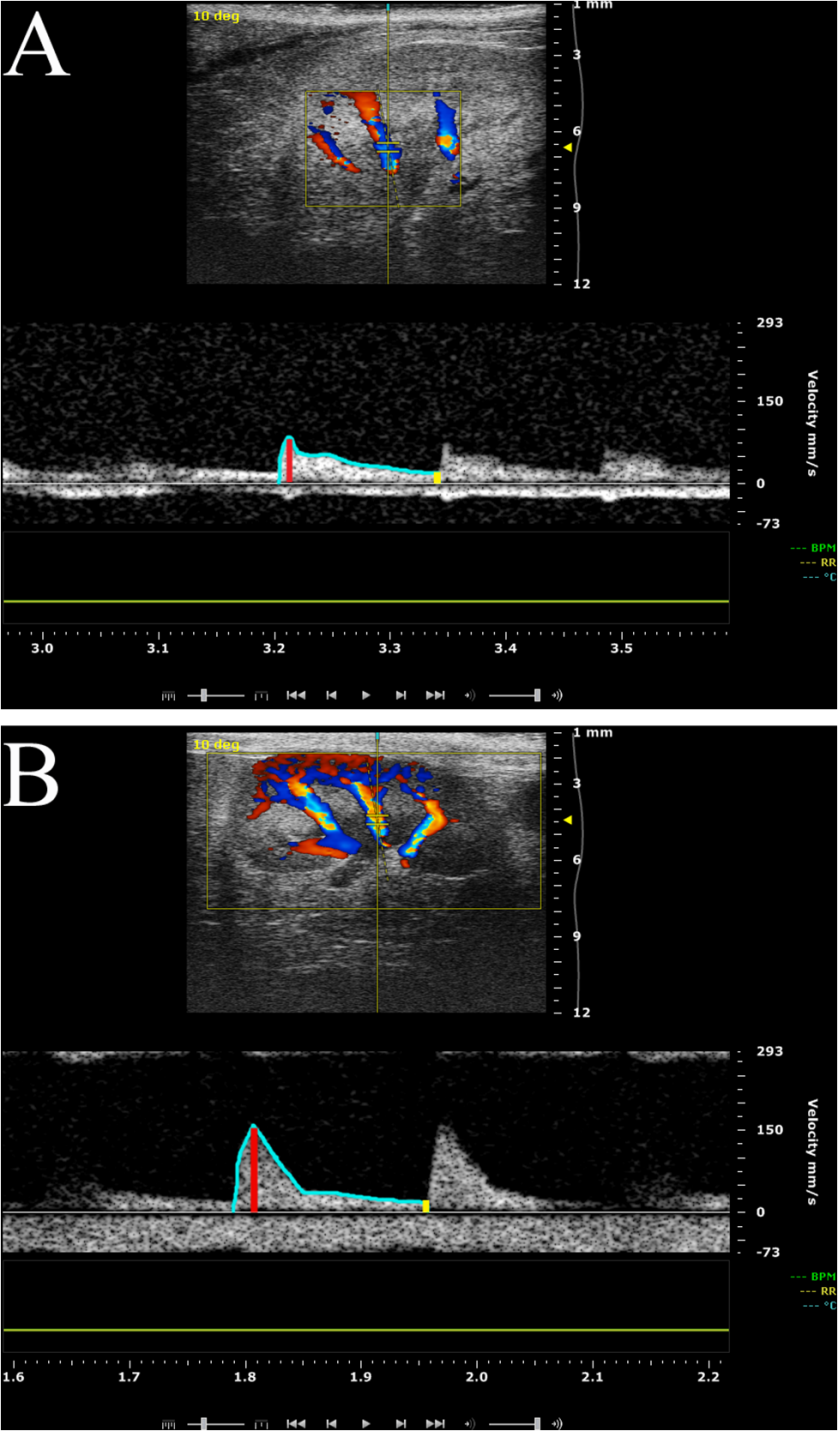
Representative color Doppler image for measurement of renal flow velocities. Typical recordings of intrarenal flow velocities measurement in segmental renal arteries were performed with color Doppler ultrasound in lean and leptin-deficient (ob/ob) mice at 21 weeks of age. The pulsatility flow profile was used for measurement of peak systolic velocity (PSV) (red line), mean velocity (MV) (blue line) and lowest diastolic velocity (LDV) (yellow line) for calculation of pulsatility index and resistive index. A: Renal flow velocity profile in lean mice. B: Renal flow velocity profile in ob/ob mice.

**Fig 3:**
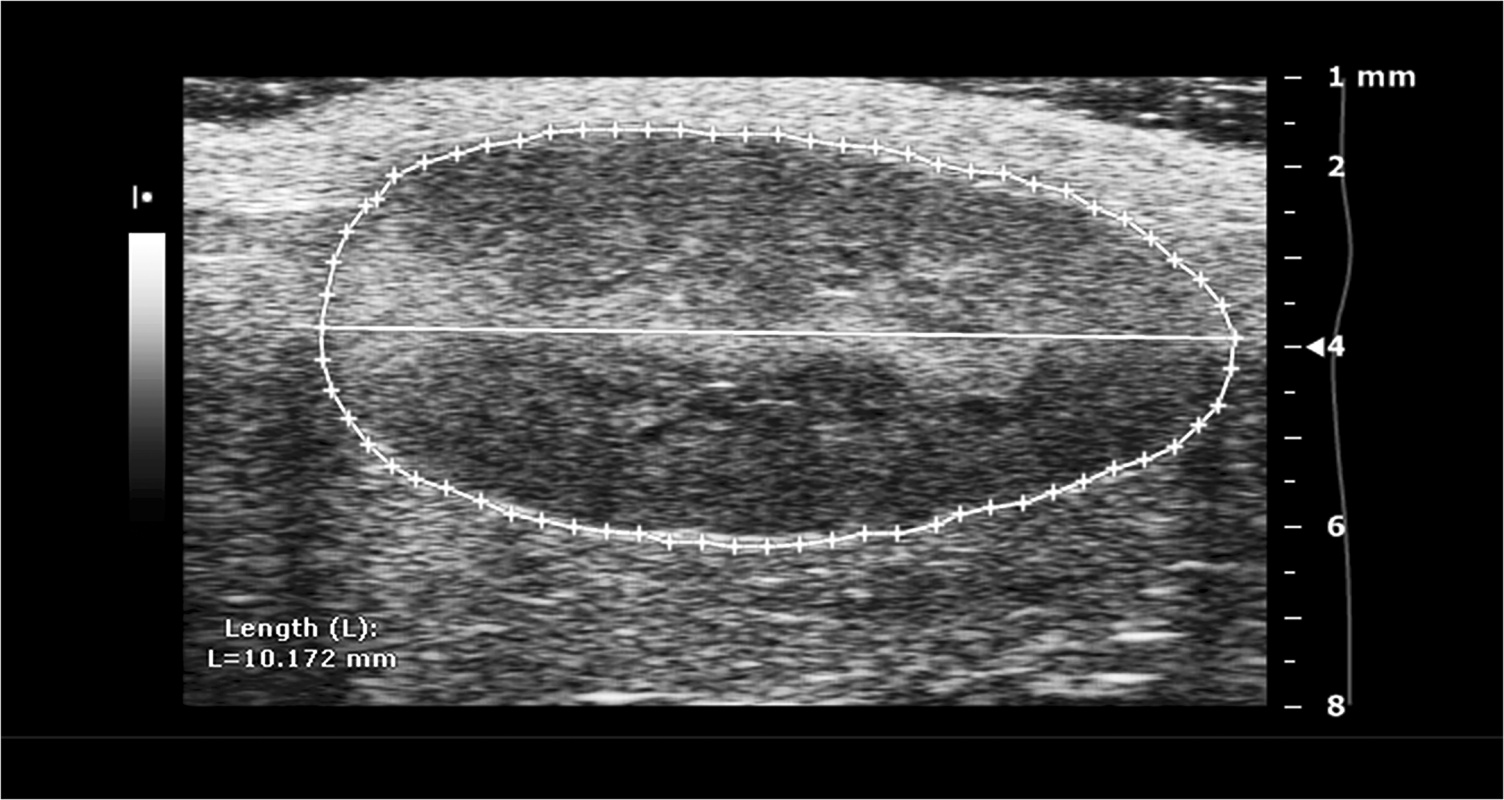
B-mode ultrasound measurement of kidney length in long-axis view. Off-line measurements of kidney length.

Ultrasound examination of the right kidney was performed at 21 weeks of age in combination with the cardiac echocardiography investigation. Standard B-mode examination of the kidney was performed in a long-axis view using the same ultrasound imaging system as described above. Following color-Doppler mapping of the renal vascular tree using a color-Doppler frequency of 32 MHz, a cursor was placed at the central segmental artery and renal flow velocity was measured using pulse wave Doppler (Fig 2). Kidney length was measured off-line at standard B-mode long axis view, blinded to the examiner (Fig 3).

The renal resistance parameters, RI and PI, were determined as follows:

RI=(PSV−EDV)/PSV

PI=(PSV−EDV)/MV

where PSV is peak systolic velocity, EDV is end-diastolic velocity, and MV is mean velocity (time averaged velocity).

### LC-MS/MS analysis of L-arginine and asymmetric dimethylarginine (ADMA) in plasma

The liquid chromatography- tandem isotope dilution mass spectrometry analysis (LC-MS/MS) of L-arginine and ADMA (Sigma-Aldrich, St. Louis, MO, USA) was based on a modified methodology previously described by E. Schwedhelm *et* al [28]. Briefly, the plasma samples were subjected to protein precipitation with acetonitrile (LC-MS grade, from Fisher Scientific Leicestershire, UK) using a Velocity 11 Bravo pipetting robot (Agilent Technologies, Santa Clara, CA). Isotopic labelled standards of the analytes, [^13^C6]-arginine and [^2^H7]-ADMA (Cambridge Isotope laboratories, Inc. Andover, MA, USA), were used as internal standards, spiked at the same level in every sample including the standards at a concentration approximately half of the concentration of the target analytes, i.e. 34 μM for L-arginine and 0.34 μM for ADMA. The precipitated samples were centrifuged at 4000 RCF for 10 min (Eppendorf Centrifuge, 5810R, Hamburg, Germany) and the supernatants were transferred to clean glass vials. Volumes of 10μL were injected onto the LC-MS/MS system comprising a UHPLC system (1290 Infinity binary pump, 1290 Infinity autosampler with thermostat and 1290 Infinity thermostated column compartment, all Agilent Technologies (Waldbronn, Germany)) coupled to a 6490 triple quadrupole mass spectrometer from Agilent Technologies (Palo Alto, CA, USA). The chromatographic separation was performed on an Accucore HILIC column (2.1x100mm, 2.6μm) (Thermo Scientific, USA) operated at 25°C with a flow rate of 0.3mL/min. The mobile phase A consisted of 100% acetonitrile and mobile phase B of 10 mM ammonium formate (pH 3.2) (Sigma-Aldrich, Steinheim, Germany). The gradient started with 30% B for 1.5min and increased linearly to 50% B over 2min, with subsequent re-equilibration with 30% B for 4min. The mass spectrometer was operated in electrospray ionization positive ionization mode.

### Quantification of vascular density through vascular area fraction

Histological staining was performed on 4μm thick paraffin-embedded tissue sections labelled with Lectin I using an IntelliPath FLX automated staining machine. Antigen retrieval was performed in a boiling citric acid solution at pH 6 for 20 minutes followed by treatment with peroxidazed I for 5 minutes to quench endogenous peroxidase activity. Nonspecific binding to tissue was blocked by incubating with rodent block M for 30 minutes prior to staining of the blood vessel with biotinylated Lectin I (B-1105, Vector Laboratories, Burlingame CA, USA, dilution 1:100) for 1 h. This was followed by 4+streptavidin horse radish peroxidase labeling for 10 minutes using 3–3’-diaminobenzidine as the chromogen. Sections were counterstained with hematoxylin. Sections where incubation with Lectin I was omitted served as negative controls. All reagents were from Biocare Medical, Concord, CA, USA.

Stained tissue sections were digitized by using a Zeiss Mirax slide scanner (3D Histech, Budapest, Hungary) to image 100% of each section. The resulting virtual slides were imported into Visiopharm Integrator System software (version 3.6.5, Visiopharm, Hørsholm, Denmark). Analytical subsections were selected, and resampled at 1.013 pixels/μm, using systematic, uniform, random, non-overlapping sampling so that 40% of each tissue slice was analyzed. Computerized image analysis was used to quantify transversely sectioned smaller vessels as follows. Subsampled images were pre-processed (using contrast, polynomial gradients and median filtering) to create various representations that enhanced relevant features (colors, edges, shapes). These images formed a training set that was annotated manually to identify tissue features for machine learning prior to pixel classification using Bayesian classification. Following feature identification and classification, post-processing steps were performed to identify blood vessels with defined characteristics: cross sectional areas between 20 and 200 μm^2^ (to avoid larger vessels, longitudinally sectioned vessels and background staining), less than 40% of the object perimeter in contact with the slide background (to avoid endothelium and tissue edge effects by selecting objects surrounded by tissue) and isoperimetric circularity values less than 8 (to select transversely sectioned vessels). These filters also excluded capillaries within glomeruli. Finally, the resulting segmentation was validated by human experts. The results are reported as the mean area fraction of the identified vessels compared to the total tissue area examined (including the vessels) for each animal as well as vessel area alone and total tissue area exclusive vessel area.

### Measurement of HbA_1c_, glucose, insulin and insulin resistance index (IR-index)

The measurement of HbA_1c_ was performed from tail vein blood at week 10, 16 and 21 using A1CNow+ (Bayer Healthcare, Austria). Blood glucose concentration was determined from tail vein using an ACCU-CHEK Compact Plus analyser (Roche Diagnostics Scandinavia AB). Blood insulin levels were measured using an ELISA kit (Ultra-Sensitive Mouse Insulin ELISA kit, Chrystal Chem Inc., IL, USA). Both glucose and insulin were measured at the age of 21 weeks, after 4 hour fasting in the awake mouse. IR-index was used to validate insulin resistance in our mice to reflect homeostatic model of insulin resistance (HOMA-IR) in humans [29]. IR-index was calculated with the following formula: fasting blood insulin (ng/mL) x fasting blood glucose (mmol/L).

Plasma levels of haptoglobin were measured using an enzymatic colorimetric method (Kit No TP 801) from Tridelta Development LTD, Ireland. Cholesterol Total was measured using an enzymatic colorimetric method (Kit No A11A01634) from Horiba ABX, France. Plasma triglycerides were measured using an enzymatic colorimetric method (Kit No 12146029 triglycerides /GB) from Roche Diagnostics GmbH, Germany. The content of triglycerides in liver and heart tissues were determined after homogenisation in isopropanaol. Triglycerides in the supernatant were measured using an enzymatic colorimetric method (Kit No A11A01640) from Horiba ABX, France. Urine Albumine was measured using a commercial ELISA kit (Cat No E-90AL) from ICL, USA.Urine Creatinine was measured using an enzymatic colorimetric method (Kit No A11A01933) from Horiba ABX, France. All mentioned biomarkers were measured at the terminal endpoint of 21 weeks of age. Albuminuria was evaluated measuring spot urine albumin/creatinine ratio (ACR, μg/mg).

### Statistics

All analyses were performed in SPSS (version 21.0, Chicago Inc, USA.) and figures were made in GraphPad Prism (version 4.03, Graphpad Inc., San Diego, CA, USA). The number of animals/group was based on previous in house experiments. A p-value of less than 0.05 was considered significant. As data appeared clearly non-normally distributed Mann-Whitney U-test was used to detect statistical differences between lean and ob/ob mice. Before analysis, we assumed an increased difference due to time in bodyweight, HbA_1c_, coronary hyperemic flow velocity, CFVR, FS and LVM between strains. Therefore a stepwise Mann-Whitney U-test on the 5%-significance level was used to compare changes between strains, starting at 21 weeks of age. In text and tables values are displayed as mean±standard deviation (SD), in graphs individual data points and mean are presented.

## Results

To study the progression of microvascular dysfunction in insulin resistant mice, we measured metabolic parameters in ob/ob mice at 10, 16 and 21 weeks. In parallel, we assessed cardiac and coronary function, followed by terminal renal vascular resistance, using the echocardiography color Doppler technique. Following these functional measures, changes related to the nitric oxide pathway was investigated. Also vascular density was determined by measuring the vascular area fraction on histological sections of the heart and kidney.

### Progression of diabetes and obesity

As expected, we found that body weight and HbA_1c_ levels were greater in ob/ob mice compared to lean controls at all three time points; 10, 16 and 21 weeks (Table 1). To determine insulin resistance, we measured fasting insulin and glucose levels solely at 21 weeks. We found that the levels in ob/ob mice were increased at this time point, resulting in an increased IR-index (Table 1).

**Table 1:** Weight data and metabolic parameters in 10, 16 and 21 weeks old lean controls and leptin-deficient (ob/ob) mice.

		Weight		Metabolic parameters				
		Body (g)	Liver (g)	HbA_1c_ (%)	HbA_1c_ (mmol/ mol)	Glucose(mmol/L)	Insulin (ng/mL)	IR-index
**10 weeks**	**Lean (n = 12)**	25±2	n.d.	4.3±0.2	24± 2	n.d.	n.d.	n.d.
	**ob/ob (n = 12)**	41±4†	n.d.	7.9±3.7	63±17†	n.d.	n.d.	n.d.
**16 weeks**	**Lean (n = 12)**	30±2	n.d.	4.5±0.3	26± 3	n.d.	n.d.	n.d.
	**ob/ob (n = 10)**	48±7†	n.d.	7.5±4.0	58±20†	n.d.	n.d.	n.d.
**21 weeks**	**Lean (n = 11)**	31±2	1.5±0.1	4.4±0.2	25± 2	10.4± 1	0.6±0.2	5.8±1.5
	**ob/ob (n = 10)**	50±9†	2.8±1.2*	7.9±4.4	63±25†	20.5±12	1.9±3.9†	40.6±8.7†

To monitor the progression of diabetes and obesity in lean and leptin-deficient (ob/ob) mice, bodyweight and glycosylated haemoglobin (HbA_1c_) in blood were studied over time. In 21 weeks old mice, wet liver weight was recorded together with glucose and insulin levels and the calculated insulin resistance index (IR-index) was determined. Statistical differences between groups in repeated data (Bodyweight and HbA_1c_) were tested using the stepwise Mann-Whitney U-test. Statistical differences between groups in remaining parameters were tested using the Mann-Whitney U-test. Values are presented as mean±SD, where

*: p<0.05

†: p<0.01.

### Coronary function, cardiac function, kidney size and renal vascular resistance

To assess cardiac and coronary function during the progression of insulin resistance in non-atherosclerotic ob/ob mice we performed sequential measurement of CFVR using transthoracic Doppler echocardiography. Basic echocardiographic data was similar between lean controls and ob/ob mice at all time-points (Table 2), while increased wet heart weight was observed at 21 weeks of age in ob/ob compared to lean mice (150±14 and 139±6 mg, respectively, p = 0.036). Also kidney length measured in ultrasound B-mode was increased in ob/ob compared to lean mice (12.4±1.0 and 11.6±0.5 mm, respectively, p = 0.027). Coronary vascular function as measured by CFVR was reduced in ob/ob mice compared to lean mice at 16 and 21 weeks of age. This was due to a reduction in the hyperemic flow velocity, since the basal flow velocity was unchanged (Table 2). Importantly, the heart rate during adenosine infusion (10 weeks: 389±86 and 345±62, 16 weeks: 373±48 and 351±51, 21 weeks: 366±41 and 332±51, lean and ob/ob respectively) was not statistically different between groups or from basal levels at any time point.

**Table 2:** Echocardiographic data and coronary flow velocity in 10, 16 and 21 weeks old lean controls and leptin-deficient mice.

		Echocardiographic data						Coronary flow velocity		
		Heart Rate (pbm)	LV ESD (mm)	LV EDD (mm)	FS (%)	LVM (mg)	LVWT (mm)	Basal flow velocity (cm/s)	Hyper-emic flow velocity (cm/s)	CFVR
**10 weeks**	**Lean (n = 12)**	380±80	3.2±0.5	4.2±0.4	24±7.3	123± 5	0.78±0.09	28± 8	61±13	2.30±0.60
	**ob/ob (n = 12)**	350±62	2.9±0.7	4.1±0.6	31±9.5	122±20	0.79±0.14	22± 7	49±13	2.30±0.60
**16 weeks**	**Lean (n = 12)**	380±60	3.2±0.4	4.2±0.3	24±8.2	129±10	0.81±0.08	27±4	67± 9	2.60±0.50
	**ob/ob (n = 10)**	370±50	2.8±0.3	4.1±0.3	31±4.8	133±12	0.85±0.09	27±5	53± 9†	2.00±0.40*
**21 weeks**	**Lean (n = 11)**	390±50	2.8±0.4	4.0±0.2	29±8.7	129±13	0.86±0.06	24± 60	61±11	2.70±0.50
	**ob/ob (n = 10)**	370±60	2.5±0.6	3.9±0.5	37±9.9	141±13	0.95±0.18	22± 90	47±15*	2.20±0.50*

Cardiac and coronary vascular function were studied in lean and leptin-deficient (ob/ob) mice over time by non-invasive transthoracic ultrasound and coronary flow reserve (CFVR). Heart rate, left ventricle end-systolic diameter (LVESD), left ventricle end-diastolic diameter (LVEDD), fractional shortening (FS), left ventricle mass (LVM) and left ventricle wall thickness (LVWT) was recorded. Coronary vascular function was assessed by measuring the coronary flow reserve (CFVR). CFVR was calculated as the ratio of coronary hyperemic and basal flow velocities. Statistical differences between groups were tested using the stepwise Mann-Whitney U-test. Values are presented as mean±SD, where

*: p<0.05

†: p<0.01.

**Fig 4:**
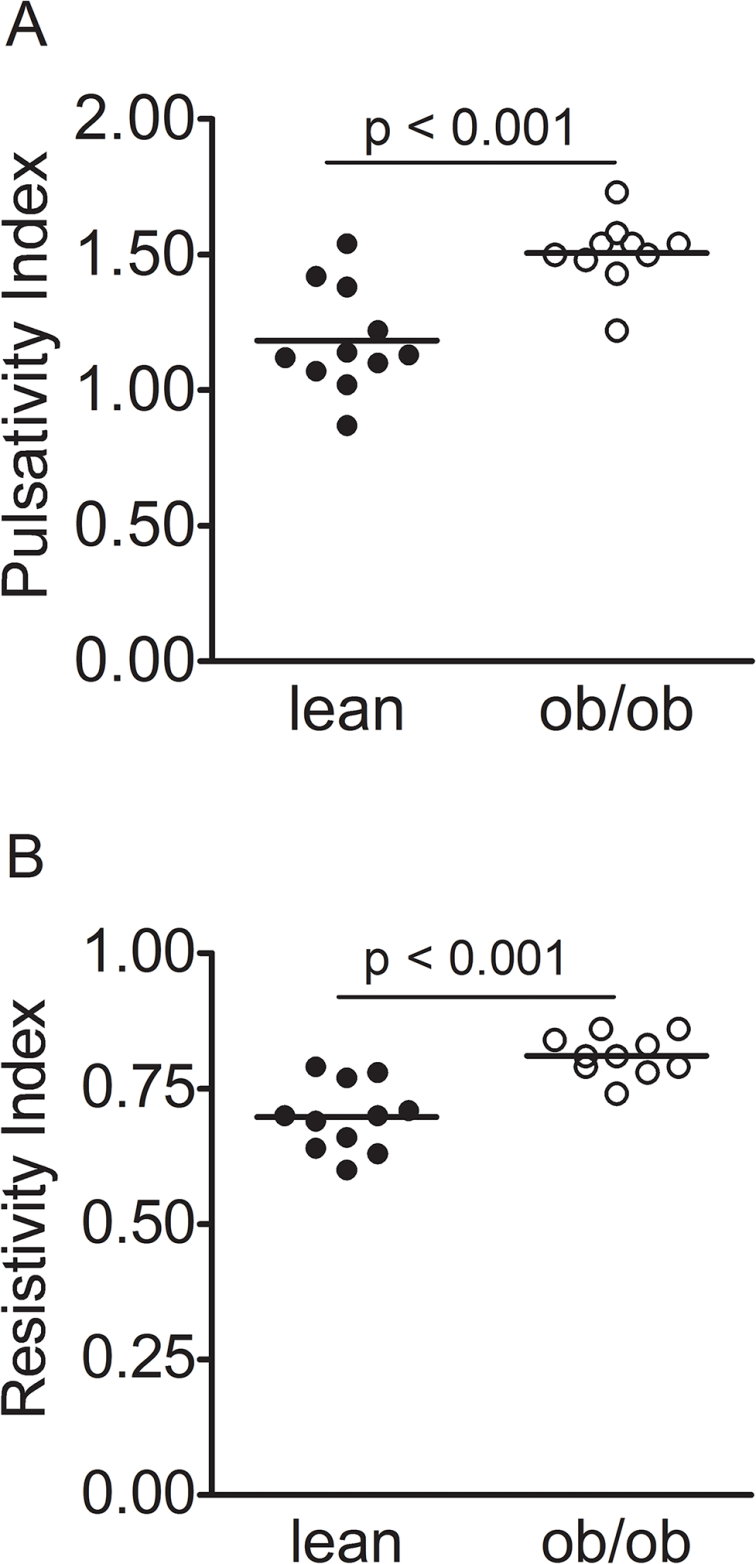
Increased renal pulsatility index and resistivity index in ob/ob mice at 21 weeks of age. Renal flow velocity was measured in lean (n = 11) and leptin-deficient (ob/ob, n = 10) mice at 21 weeks of age using the ultrasound color Doppler technique. A: Renal pulsatility index, B: Renal resistivity index. Values are presented as individual datapoints and means. Statistical analysis was performed using Mann-Whitney U-test and a p-value of less than 0.05 was considered significant.

To non-invasively measure renal vascular resistance, we applied a Doppler-guided imaging protocol where PI and RI were used. The ob/ob mice displayed increased mean PI values (1.50±0.13 and 1.18±0.19, respectively) as well as increased mean RI values (0.81±0.04 and 0.69±0.06, respectively) when compared to lean mice (Fig 4).

Furthermore, ob/ob mice compared to lean mice also displayed a 3-fold increase in spot albumin/creatinine ratio (ACR) in urine (Table 3).

**Table 3:** Plasma and urine biomarkers at 21 weeks of age in lean controls and leptin-deficient mice.

		L-arginine (μM)	ADMA (μM)	L-arginine/ ADMA	Haptoglobin (g/L)	Albumine/ Creatinine Ratio (μg/mg)
**21 weeks**	**Lean (n = 11)**	68±28	0.68±0.09	101±37	0.2±0.1	18± 3
	**ob/ob (n = 10)**	15±28*	0.76±0.11	18±32*	0.8±0.7	59±17*

Lean controls and leptin-deficient (ob/ob) mice were studied at 21 weeks of age regarding functional endothelial data. Plasma concentration of L-arginine and asymmetrical dimethylarginine (ADMA) were analysed and L-arginine/ADMA ratio was calculated. Plasma levels of Haptoglobin were analyzed as well as spot urine albumin/creatinine ratio. Statistical differences between groups were tested using the Mann-Whitney U-test. Each value is presented as mean ± SD where

*; p<0.001.

### Blood biomarkers

To investigate the nitric oxide pathway we measured L-arginine and ADMA in plasma. We found significantly lower plasma levels of L-arginine (Table 3) in ob/ob mice compared to lean controls at 21 weeks of age, indicating dysfunctional nitric oxide pathway, while no difference was seen in ADMA levels (Table 3). The L-arginine/ADMA ratio was significantly lower in ob/ob mice compared to lean mice (Table 3).

No difference was seen in plasma acute phase protein haptoglobin (Table 3), total cholesterol (3.6±1.9 and 2.8±0.4 mM, respectively) or triglycerides (1.5±1.3 and 0.8±0.3 mM, respectively). However, triglyceride levels in liver tissue were markedly higher in ob/ob mice compared to lean mice (15.2±13.5 and 3.9±3.1g triglycerides/100g liver tissue, respectively, p = 0.013) as well as triglyceride levels in heart (1.1±0.3 and 0.3±0.1g triglycerides/100g heart tissue, respectively, p<0.001).

### Cardiac and renal vascular area fraction

**Fig 5:**
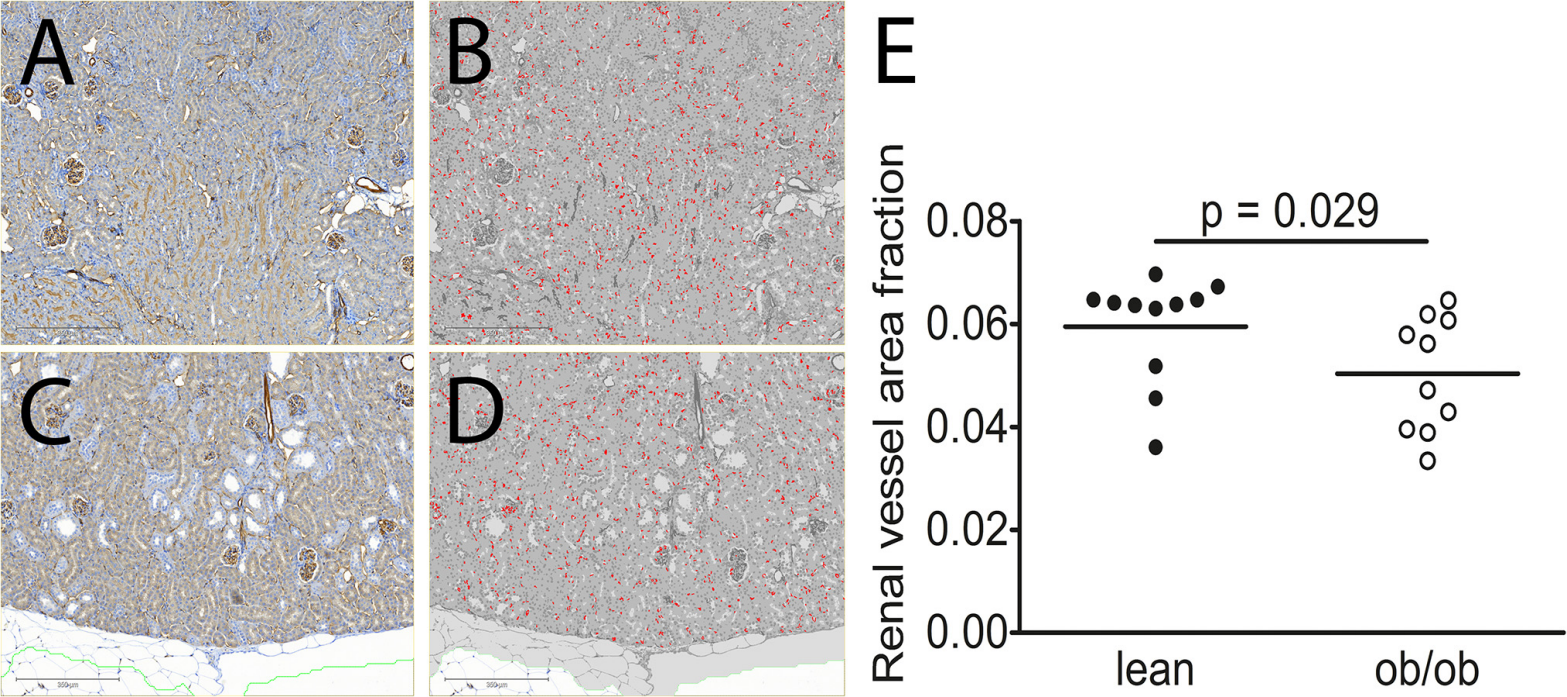
Reduced renal vessel area fraction in ob/ob mice at 21 weeks of age. Renal vessel area fraction was measured in lean (n = 11) and leptin-deficient (ob/ob, n = 10) mice at 21 weeks of age. A-D: Representative samples of kidney sections immunostained with Lectin I. A: Lectin I in lean mice. B: Computerized image analysis in lean mice. C: Lectin I in ob/ob mice. D: Computerized image analysis in ob/ob mice. E: Renal vessel area fraction was calculated as the mean area fraction of the identified small vessels compared to the total kidney tissue area examined. Values are presented as individual datapoints and means. Statistical analysis was performed using Mann-Whitney U-test and a p-value of less than 0.05 was considered significant.

To study structural vascular changes we measured cardiac and renal vascular area fraction. When comparing cardiac vascular area fraction in ob/ob mice (0.026±0.005) with lean mice (0.027±0.005), no statistical significant difference was seen. However, renal vascular area fraction was reduced in ob/ob mice (0.049±0.012) when compared to lean mice (0.059±0.011; p = 0.029; Fig 5). Interestingly, the actual vessel area was not statistically different in ob/ob mice (1155881±371932**μ**m^2^) compared to lean mice (1104850±272247**μ**m^2^). However, the total renal tissue area exclusive vessel area was increased in ob/ob mice (21514595±2875745**μ**m^2^) compared to lean mice (17331054±2186404**μ**m^2^), p = 0.002.

## Discussion

The present study confirms impaired glucose homeostasis with increased levels of insulin, glucose, HbA_1c_, as well as IR-index in ob/ob mice compared to lean controls. We demonstrate that ob/ob mice show cardiac microvascular dysfunction from the age of 16 weeks. Interestingly, using a renal imaging protocol, we detected increased renal vascular resistance in the ob/ob mice at 21 weeks of age.

Since ob/ob mice do not develop atherosclerosis [24], assessment of CFVR in this model should thus be a measure of microvascular function. CFVR has been shown to be a measure of coronary function in the absence of obstructive plaques and is partly mediated via NO-dependent pathways [30]. In our study, we detected a statistically significant decrease in CFVR in the ob/ob mice starting at 16 weeks of age and present at 21 weeks of age compared to controls. Consistent with our findings, in a recent study in obese patients without obstructive CAD, decreased CFR was observed compared to lean individuals. This indicates impaired microvascular function and supports the translational relevance of the model [31]. We have shown previously that CFVR and CFR are well correlated and that the diameter of epicardial arteries is limitedly increased during adenosine-infusion [27], thus a lowered CFVR in ob/ob mice should reflect a reduced coronary volumetric flow reserve. The reproducibility of CFVR in mice has been validated in our lab with acceptable low inter- and intra-observer variability [27]. Also, since measurement of cardiovascular function is highly dependent on heart rate [26], we succeeded to perform all measurements keeping heart rate similar between lean and ob/ob mice both at basal and hyperemic conditions to avoid this obvious confounding factor.

CFVR has been shown to be impaired in patients with type 2 diabetes with negative coronary angiogram [6,7] and demonstrated to have a strong prognostic value for future events in this patient group [32]. Katz et.al. demonstrated decreased CFR and inward hypertrophic remodeling of coronary arteries in a mouse model of type 2 diabetes, the leptin receptor deficient db/db mouse [13]. Furthermore endothelial dysfunction was observed in mouse coronary arterioles measured *ex vivo* [12]. We have previously shown that arginase activity was increased in a non-atherosclerotic rat model of type 2 diabetes, which resulted in impaired coronary artery function i.e. when measured as CFVR using high-resolution color Doppler ultrasound [17]. In the same study, the impairment was restored by acute treatment with an arginase inhibitor resulting in a shift in the pathway for the substrate L-arginine that increased NO-bioavailability. L-arginine is the substrate for both nitric oxide synthase (NOS), which converts arginine to NO and citrulline, as well as for arginase that converts L-arginine to urea and ornithine. Arginase competes with NOS for L-arginine and thereby might contribute to the decreased NO-availability in pathological conditions such as type 2 diabetes [33]. Our present study demonstrates decreased levels of plasma L-arginine in the type 2 diabetic ob/ob mice compared to lean controls. This might lead to a lack of NO-availability and thereby reduced coronary artery function. In line with our observations, Saraiva et.al. demonstrated decreased cardiac NO production and increased oxidative stress in ob/ob mice [34]. Endothelial dysfunction due to decreased NO bioavailability caused by hyperglycemia and insulin resistance are of known importance. Another possible contributor is leptin deficiency. Winters et. al. showed that endothelial dysfunction in ob/ob mice can be reversed by leptin replacement [11] and could therefore not be excluded as an contributor to the observed micorvascular dyfunction in this study. However, endothelial dysfunction has been observed also in non-obese type 2 diabetes models [17] and insulin-induced relaxation was significantly decreased in the ob/ob aortas [14]. The present hyperglycemia and insulin resistance are thus likely to be contributing to the pathophysiology of the vessels. Furthermore, hypertension is known to be associated with endothelial dysfunction and present in the ob/ob model at 24 weeks of age, but not at the level of a metabolic syndrome model [15]. In the published study [15] the observed hypertension in the ob/ob mice was compared with wild type C57/6 mice, while in the present study lean mice were used as controls. We did not observe increased mean arterial blood pressure in ob/ob mice at 21 weeks of age, measured using tail-cuff in anesthetized mice (data not shown).

At a relatively young age, ob/ob mice do not seem to develop overt systolic contractile dysfunction, even though there is conflicting data in the literature [35,36]. However, impaired diastolic function has been reported previously [36], this may partially be explained by increased left ventricle mass. In our study wet heart weight of ob/ob mice was increased compared to lean mice which could indicate an increased left ventricle mass. Moreover, the ob/ob cardiomyocytes have been shown to be profoundly insulin resistant. Impaired insulin action in the heart is associated with a metabolic profile in which glucose utilization (glycolysis and glucose oxidation) is reduced and fatty acid utilization increased [37]. Leptin-deficient ob/ob mice hearts exhibit a fixed defect in glucose oxidation in response to insulin or to changes in delivery of fatty acids. Furthermore, increased fatty acid oxidation rates and myocardial oxygen consumption, together with increased mitochondrial uncoupling that has been shown in the ob/ob mice, may explain decreased cardiac efficiency, especially during stress [36–38]. In agreement with previous studies we found accumulation of triglycerides in the hearts of ob/ob mice [36]. All of these above described mechanisms could also partly explain the decreased CFVR observed in this study in addition to the abnormal NO-pathway. Furthermore, insulin resistance as well as overall obesity could be features of nonalcoholic fatty liver disease, and the ob/ob mice in our study, as previously known display increased triglycerides in the liver.

It has been shown that renal vascular resistance is greater in CKD of diabetic origin than other etiologies, which may be explained by increased systemic arterial stiffness in diabetic subjects [20]. Interestingly, in the present study we show increased resistance in the renal vascular bed in the ob/ob mice as measured by increased RI and PI in the segmental renal artery. The underlying cause is most likely multifactorial, including the observed decreased L-arginine/ADMA ratio, which could lead to decreased NO-dependent vasodilatation. This is in line with the renal microvascular perfusion dysfunction seen in non-atherosclerotic diabetic rats [39]. Furthermore, we detected a lowered vascular density as measured by relative vessel area in the kidneys from ob/ob mice. Interestingly, there was an increase in total renal tissue area rather than a decrease in the actual vessel area, indicating initial renal hypertrophy which also was verified by ultrasound measured increased kidney length in the ob/ob mice. This might lead to increased resistance in the renal tissue, generating the observed high-resistance flow profile in ob/ob mice. In line with our data, others have shown 1.2x increase in kidney weight in ob/ob mice compared to controls [40] as well as that the ob/ob mouse model expresses alterations in the arteriol network of skeletal muscle [41].

The ob/ob mice showed a small increase in spot urine ACR, which further may indicate initial renal pathological changes without histological evidence e.g. matrix expansion, podocyte injury nor interstitial fibrosis (unpublished data) of manifested diabetic nephropathy. Furthermore, elevated resistance in renal blood flow in type 2 diabetic patients has been shown be a marker of early renal vascular alteration, even before the onset of microalbuminuria [42]. In line with this data, the ob/ob mouse model might be suitable for further mechanistic studies of endothelial dysfunction as well as for changes in renal blood flow as an early marker of renovascular changes.

Cardiovascular and renovascular diseases are known to be closely related. Patients with non-obstructive CAD and CKD have lower CFR than those with normal glomerular filtration rate (GFR) [8] and low CFR is of prognostic value for CV outcome in patients with CKD [43]. The fact that ob/ob mice display coronary- and renovascular dysfunction probably at a microvascular level, together with a mild diabetic metabolic phenotype makes it an interesting translational model for further preclinical mechanistic and interventional studies. Finally, the non-invasive techniques used in the current study seem to be highly feasible for longitudinal studies in pre-clinical research, thus in a beneficial way reducing the number of animals used.

## Limitations of the Study

The ob/ob mouse is a type 2 diabetic model due to leptin-deficiency, although it does not develop diabetic nephropathy. The clinical relevance of the specific gene mutation is of minor importance, however, the pathological consequence of insulin resistance on endothelial and microvascular dysfunction is still of relevance. The model also lacks atherosclerosis, which is commonly seen in the clinical setting. Although, in support of the ob/ob mouse model, vascular function in the absence of obstructive coronary artery disease is also of known significance in this patient group. Due to previously reported potential direct effect on blood glucose during isoflurane anesthesia, we always measure glucose through blood sampling in conscious mice in our lab. Using this approach, potential stress-related glucose elevation cannot be excluded, which may explain the slightly higher glucose level at 21 weeks of age compared to literature data. However, since all mice were treated in a similar way, we still think that comparison between groups is valid. Also, although all offline measurements were performed blinded to the examiner, due to obvious weight difference between lean and ob/ob mice the ultrasound examination could not be performed blinded, which adds a degree of subjectivity in flow profile registration. The authors are aware that the ob/ob mouse model in addition to being a model of type 2 diabetes, is also a model of obesity. Thus, the relative contribution of each of these risk factors to the observed cardiac and renal vascular dysfunction cannot be ruled out in this present study.

## Supporting Information

S1 ARRIVE Checklist ARRIVE Guidelines Checklist. (PDF)
